# Wearable Technology to Increase Self-Awareness of Low Back Pain: A Survey of Technology Needs among Health Care Workers

**DOI:** 10.3390/s21248412

**Published:** 2021-12-16

**Authors:** Andrea Ferrone, Christopher Napier, Carlo Menon

**Affiliations:** 1 Menrva Research Group, Schools of Mechatronic Systems & Engineering Science, Simon Fraser University, Vancouver, BC V5A 1S6, Canada; ferrone.andrea@gmail.com (A.F.); cmenon@sfu.ca (C.M.); 2 Department of Physical Therapy, Faculty of Medicine, University of British Columbia, Vancouver, BC V6T 1Z3, Canada; 3 Biomedical and Mobile Health Technology Laboratory, Department of Health Sciences and Technology, ETH Zurich, 8092 Zurich, Switzerland

**Keywords:** safety, risks, human factors and ergonomics, wearable technologies, musculoskeletal injury, cross-sectional survey, nurses, machine learning, health and social services, random forest

## Abstract

Low back pain (LBP) is a leading contributor to musculoskeletal injury worldwide and carries a high economic cost. The healthcare industry is the most burdened, with nurses, in particular, being highly prone to LBP. Wearable technologies have the potential to address the challenges of monitoring postures that contribute to LBP and increase self-awareness of workplace postures and movements. We aimed to gain insight into workers’ perceptions of LBP and whether they would consider using wearable monitoring technologies to reduce injury risks. We conducted a cross-sectional survey to gather information from a selected population of nurses. Sixty-four participants completed the survey, and data were analyzed with the support of Machine Learning techniques. Findings from this study indicate that the surveyed population (64 nurses) is interested in these new approaches to monitor movement and posture in the workplace. This technology can potentially change the way ergonomic guidelines are implemented in this population.

## 1. Introduction

The World Health Organization recently published a report stating that “Musculoskeletal conditions are the leading contributor to disability worldwide, with low back pain being the single leading cause of disability globally” [1]. Low back pain (LBP) is ranked sixth in terms of overall burden worldwide [2].

Due to the idiopathic nature of LBP, it remains one of the most underestimated musculoskeletal conditions and is often not compensated as a cause for disability [3,4]. However, the economic effects of LBP on society are clear. In North America, the cost of LBP amounts to more than USD 100 billion per year [5,6], with up to 60% of this amount being a consequence of lost wages and productivity.

Low back pain is common among working populations worldwide. The Association of Workers’ Compensation Boards of Canada calculates that the majority of lost-time claims are due to traumatic injuries and disorders (88.3%), which includes muscles and tendons injuries. The claims associated with the trunk contributed 35.6% of that amount [7]. Most of these claims come from the health and social services industry (18% of total claims). Within this sector, nurses, in particular, are prone to LBP as their daily work requirements involve patient handling and repositioning, as well as many tasks involving bending, twisting, pushing and pulling [6,7,8].

Despite the prevalence, the etiology of LBP is still not fully understood. The topic is complex to study since, in many instances, the causes for LBP are unclear and are rarely directly linked to a definitive issue or condition [8]. In addition, many factors, such as worker history, individual variability in load distribution on the spine, and the lack of a clear gold standard for measurements across studies, make it challenging to conduct reliable clinical studies with participants. A review conducted by Saraceni et al. [9] highlighted that even the task of lifting weight improperly while bending or not bending at the waist could not be directly correlated with low back pain. Specifically, they reported that a strict correlation is not possible because the quality of the literature under examination—though vast at more than 2300 papers—is too low to provide clear evidence of correlation.

On the other hand, cadaveric studies, which have looked directly at the mechanical structure of the spine, have been able to provide more reliable data and clearer rationale for the movements or postures that might lead to back pain or injury [10,11]. Spine loads are usually classified as compressive forces (compress the intervertebral discs along the vertical axis of the spine), shear forces (acting along the transverse plane of the intervertebral discs), or torsional forces (rotational forces applied around the longitudinal axis of the intervertebral discs) [12,13]. Cadaveric studies also provide the basis on how to move to reduce the stress on the spine, which may reduce the risk of developing LBP. For instance, in order to withstand loads optimally, it has been suggested that the spine should maintain a neutral curvature (thoracic kyphosis and lumbar lordosis) during sustained postures or while performing lifting actions [14]. The literature shows that lifting heavy or bulky objects creates compressive spinal forces that may lead to pain or injury [15,16]. The gravitational force on the trunk while bending or pushing, and pulling can generate an overload of shear forces if it is not equally distributed via the spine’s neutral “S” shape [17].

As a result of these cadaveric studies, many prevention and intervention approaches have targeted poor postures and movement patterns [18,19,20,21]. Nurses working in long-term care spend approximately 25% of their time flexed beyond 30°, with peak flexion angles greater than 75° in many tasks [22]. Most of the activities throughout a shift do not involve lifts or transfers but are spent performing housekeeping, administrative, and patient care activities. Therefore, monitoring postures throughout the entire working shift is necessary when assessing injury risk [22].

Many ergonomic factors can contribute to LBP [23,24,25,26,27,28,29,30,31]. Jones et al. suggested four categories of risk factors: genetic traits such as a worker’s predisposition to injuries; morphological traits such as a worker’s vulnerability to injury; psychosocial traits such as a worker’s susceptibility to injury; and biomechanical aspects [32]. The biomechanical aspect is easily quantifiable and can be addressed through ergonomic recommendations and education regarding techniques to make the workplace environment safer [33].

Training and local government guidance are in place for worker categories considered at risk of injury [34]. ISO regulations are also in place for industries to reduce the risk of injuries, even if they are often vague and incomplete [35,36]. Assessments such as the Rapid Upper Limb Assessment (RULA) [37] or the Revised NIOSH Lifting Equation [38] are used to quantify and manage risks of LBP in the workplace. Despite these efforts, it is unknown how closely these guidelines are being followed in the workplace, as it is challenging to monitor constantly changing postures and movements throughout the day. The evidence provided by the numbers of claims for LBP [5] suggests that workers are not following guidelines or the procedures are underestimating the number of risks they face.

There needs to be a shift in the approach to monitoring movements and postures in the workplace that might lead to more successful injury prevention strategies. There is, therefore, a need for monitoring technology in the workplace to increase self-awareness to reduce the risk and incidence of low back injuries. Several cutting-edge technologies could be used to monitor back posture. Some of these are small as a cotton thread and could be seamlessly implemented into textiles to monitor movements and performance [39]. Others, such as Inertial Measurement Systems [40], are already well established and more reliable such as BackTone posture corrector [41], Kinetic Reflex Smart Wearable [42], Prana wearable device [43], Alex Wearable device [44] and Life-Booster [45]. Finally, some technologies, such as electrical impedance tomography or force myography, could also monitor the load and work of the muscle-tendon system during movements and postures [28,29]. All of the above could be implemented to establish a robust monitoring system for workers at higher risk of injury, with the potential for feedback and warnings to be sent to workers and employers.

Different studies and clinical trials provide evidence of the effectiveness of wearable technologies for minimizing hazardous postures during daily living activities [22,46,47,48,49]. Even though the research provides evidence of the usefulness of wearable devices, it is still uncertain if workers will adopt the technology for daily use. Wearable technologies require high adherence and commitment from the user. A device that is not used regularly or adequately will not provide the desired outcome. Furthermore, other factors can be a barrier to the adoption of wearable technology (e.g., privacy concerns, peer influence, functional congruence, effort expectancy, etc.) [50].

The purpose of this study was to gain insights into workers’ perceptions of LBP and whether they would consider the use of wearable monitoring technologies to increase their awareness and reduce injury risks. Specifically, a survey to investigate if novel technologies could be introduced in the workplace was designed to determine the need for new technology among nurses and understand which technology could be used based on how much workers are willing to pay for a solution.

## 2. Materials and Methods

### 2.1. Design

A cross-sectional survey was designed to gather information from a selected population of health and social service workers. Specifically, a purposive sampling method was chosen, and nurses were selected as the target population [51].

The survey was developed through an iterative question design process between researchers and a panel of nurses. The research team discussed the number of questions, their relevance and sequence, the language used, and the simplicity of each question. Particular care was taken to design the script for each question to avoid any influence of leading questions or bias from the research team [52,53]. A panel of three volunteer nurses was then consulted to review the survey. Further modifications were made to change the language and clarity of the questions. The survey was designed for the online platform provided by Google Forms.

The survey was composed of 21 questions, 16 of which were multiple choice, and 5 were short answer open questions. The estimated time to complete the survey was 10–15 min. The questions were grouped into four major topics: nursing demographics, history of LBP, perceptions/opinions on LBP, and opinions on technological solutions to LBP. A summary of the questions divided by topic is presented in Table 1.

### 2.2. Participants

A purposive nonprobabilistic sampling method was used to recruit 64 participants. The participants were recruited using social media platforms and via third-party recruitment using an email script and word of mouth. A link to the survey was sent to personal contacts of the research team who are nurses, posted on social media, or emailed a survey link. A cover letter, email script and flyer were prepared to introduce the survey, its objectives, and the research.

The recruited participants included registered nurses, nurse practitioners, nursing assistants, nursing aides, nursing students, and nursing managers of at least 19 years of age working in hospitals, long-term care facilities, home care, hospices, or other care facilities in the greater Vancouver area, British Columbia, Canada.

### 2.3. Ethical Considerations

The Office of Research Ethics at Simon Fraser University approved the study protocol and survey, and all participants gave informed consent prior to participating. The objectives and voluntary nature of the study were explained to participants. Confidentiality was assured, as no identifying information was collected during the survey. Anonymized survey data was sent to a private Google Drive account of the laboratory and stored on password-protected computer hard drives and a secured server at Simon Fraser University. Participants were also informed that during the online survey, anonymized data could be stored on a Google server located outside of Canada.

### 2.4. Data Analysis

Completed surveys were collected and printed. All copies were then divided randomly into three piles. A different team member subsequently digitized and organized the answers from each pile in Microsoft Excel (Microsoft Corporation, Redmond, DC, USA) spreadsheets. The team leader later merged the three files into a single Excel document. This process ensured that the risk of participant identification was practically null. In Excel, data was organized by pivot graphs to combine multiple results for further analysis.

It was hypothesized that the pool of answers could hide a repeatable behaviour pattern. One of the study’s aims was to reveal the pattern (i.e., the set of answers) specific for the nurses likely to adopt technology for LBP. Potentially, this pattern can be decrypted by adopting advanced Machine Learning (ML) algorithms. The parameter *Price* was selected as the value to rate nurses’ likelihood to adopt the new technology. To simplify, the parameter *Price* was divided into two classes: less than CAD 49 (class = 0) and more than CAD 50 (class = 1).

Python Programming language (Python Software Foundation, Delaware, USA) and Jupyter NoteBook (Jupyter notebook is an open-source web application that allows the user to create and share documents that integrate live code (https://jupyter.org/ accessed on 10 December 2021)) were used to create the models. Specifically, “Seaborn” and “Sklearn” libraries [54,55] were used to generate a heat map for feature correlation and to run Random Forest (RF) classification analysis. Files were converted into a data frame as an ML input [56]. Participants with incomplete answers were removed from the data frame. This decreased the dataset from 64 to 58 participants. Multiple answers were converted to numerical classes (e.g., yes = 1, no = 0 and, so on). Price was considered the output “Y” for the RF classification and a set of 11 questions were used as features for the models.

To create the models is necessary to have a set of data for training (i.e., create the model) and a second for testing (i.e., evaluate its accuracy). For such purposes, we randomly split the data into two sections, one for training the model and one for testing it. Two different techniques were used to split the data and compare the results. The first technique was based on a random split of 80 and 20. Specifically, the first 80% was allocated for training and the remaining 20% for testing. The second technique was based on Cross-Validation (CV) [57]. The results of both methods were used to assess the quality of the created model.

Random Forest was preferred over other algorithms, as the nature of the decision tree structure is naturally more adaptive to human behaviour, and the fact that it is one of the two most used models for survey research [58]. The classification was selected over regression since the study’s aim was only to obtain an indication of the population trend (classification) and not an exact number for the price aspect (regression).

The confidence level and margin of error for the survey were computed by an online service platform [59]. Based on the Canadian Nurses Association, when the survey was administered, the total population of regulated Nurses in Canada was 431,769 [60]. Relying on these statistics, the computed confidence level for the survey (64 participants) was 90%, with a margin error of about 10%.

## 3. Results

### 3.1. Demographics

Initially, 82 subjects participated in the survey. Following the study aim, 17 participants were excluded as they did not identify as part of the targeted worker groups. Of the remaining 64 participants, 71% completed the entire survey, 23% skipped at least one question, and 6% skipped two questions. The open short questions were skipped 62% of the time. Gender and experience demographics are shown in Table 2.

The population surveyed was mainly employed (82%) in a single institution (Long term facilities: 62%, Hospitals: 20%); the remaining 18% were working in more than one facility (community and public health).

Part of the study goal was to understand how often high-risk tasks for the back were performed by the healthcare workers during a typical shift. One category of tasks often reported as potentially high risk was patient handling (e.g., repositioning, lifting, transferring) [61,62,63]. Thus, frequency of patient handling was categorized as Often performed (six or more patient handlings per shift), Occasionally (one to five patient handlings per shift), or Rarely (less than once per shift). We found that 41% were in the Often category, 36% fell into Occasionally and 23% in Rarely. This indicates that 77% of the surveyed participants were strongly involved in patient handling.

### 3.2. History of LBP

To investigate the correlation between a history of LBP and the other survey responses, participants were asked whether they were conscious of their back condition. Thirty-three percent indicated that they currently feel back pain, although not associated with any activity or event, whereas 19% indicated that they believe their pain is due to incorrect body postures or movements. Another 19% directly associated their back pain with work activities, and 6% associated back pain with sporting activities. Further, 11% of the surveyed population worked with diagnosed back conditions (e.g., back injury or chronic back pain), 9% did not have any back issues, and 2% reported multiple reasons for their back pain (e.g., work, sport, bad posture habits). Agglomerated results indicate that 89% of all respondents worked with back pain or back conditions.

### 3.3. Strategies and Solutions

**Figure 1:**
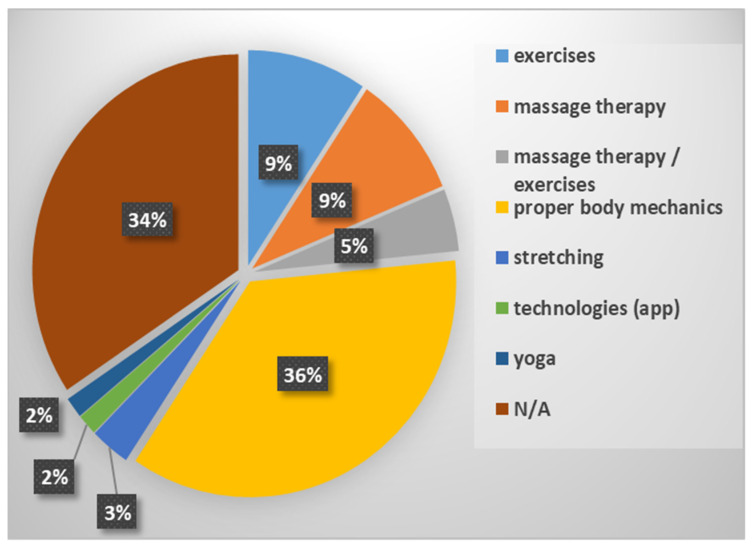
Strategies and solutions adopted.

The third section of the survey focused on strategies or solutions used by nurses to mitigate or prevent LBP. It was found that 64% adopted a strategy or solution, and within this percentage, 34% found it helpful, while 30% considered it very helpful. However, approximately one-third of the respondents did not take action. The distribution of the strategies or solutions adopted is summarized in Figure 1.

**Figure 2:**
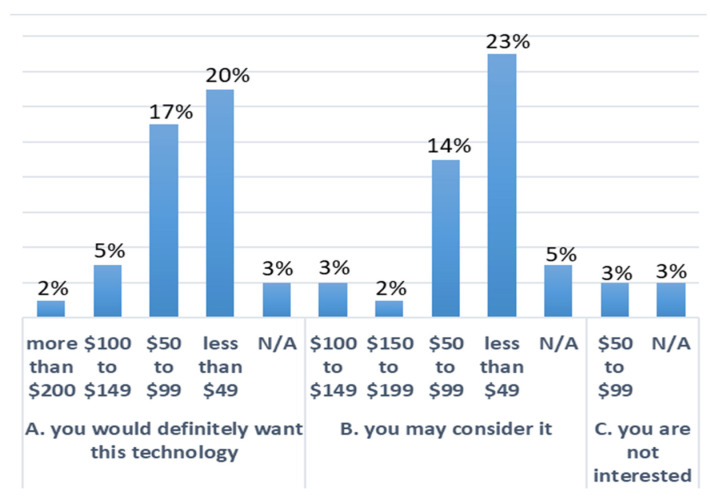
Association between price (CAD) and interest in new technology divided into three categories (A, B, C). The percentages represent the frequency of the answers.

**Figure 3:**
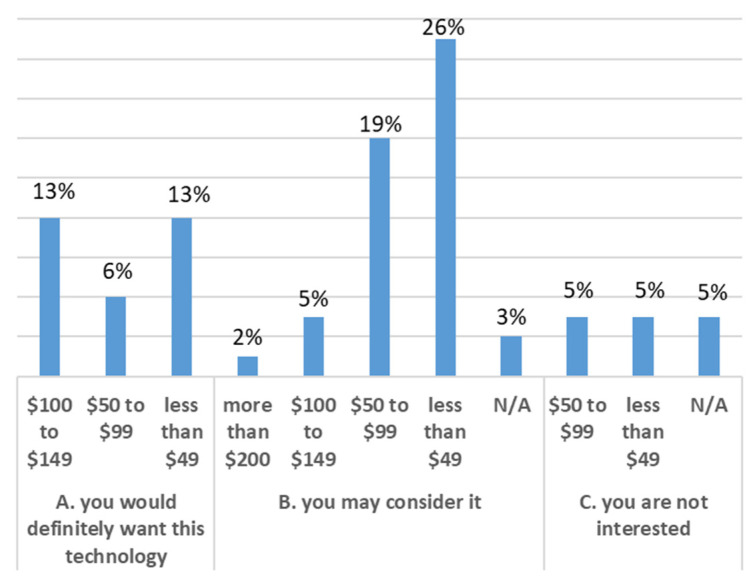
Association between the price (CAD) for new features and interest in new technology divided into three categories (A, B, C). The percentages represent the frequency of the answers.

Our investigation included an assessment of how respondents would accept a hypothetical technology that could monitor movements of the back and provide feedback to help improve posture and decrease the likelihood of LBP. The results highlighted that 47% of the participants would definitely want to adopt the technology. In comparison, another 47% were open to considering the adoption of the technology, with only six percent not interested in the proposed solution. When asked about the reason for these answers, 50% replied that it would be nice to have such technology available. Another 22% indicated they need it, while 19% said they like new technologies. The rest selected “I do not need it”, and 1% skipped this question. Finally, respondents were asked how much they would be willing to pay for such technology. The results are summarized in Figure 2. It was investigated whether increasing the features of the hypothetical device would increase the interest of the respondents. Specifically, respondents were asked if they would change their answer if the technology could also give feedback about mobility (e.g., step count, time spent in a sitting position versus a standing position, etc.). It was discovered that the percentage of participants that would want to adopt the technology decreased to 31%, whereas the category “I may consider it” and “Not Interested” increased to 55% and 14%, respectively. Respondents were then asked how much they would be willing to pay for the hypothetical technology with more features. The results are shown in Figure 3.

### 3.4. Technology and Solutions Opinions

Participants were asked to suggest features and give their opinion on what information they would like to receive from a new technology that assesses postures and movements with a series of five questions (three short open questions and two multiple choices).

**Figure 4:**
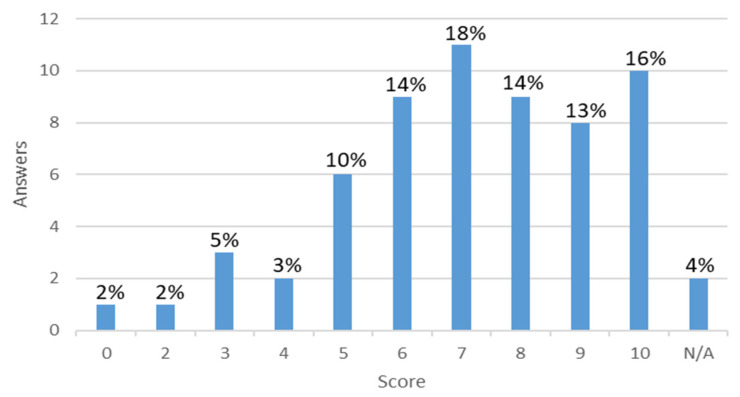
Score from zero to ten on how likely they would recommend the technology. The percentages represent the frequency of the answers.

Eighty-three percent of respondents favoured a technology that could document a back injury as work-related, while 14% were against it. Of the 83% in favour, the most common reason given was that it could help for insurance claim purposes (20%), and 17% stated it could help better understand the source of the problem. Only a small percentage (2%) were hesitant about privacy. Of those who were against it, again, only three percent were concerned about the privacy of their personal information, while the remainder did not specify why. Another short open-answer question gave us a range of possible features that the population would like to implement in a hypothetical technology. Sixty-four percent of respondents mentioned that they would want alerts about body alignment, bad postures, or strain risks, 16% highlighted the need for step counts, and five percent wanted a biomechanical representation of their movements. Other suggestions by the respondents are presented in Table 3. Respondents were also asked how likely (on a numerical rating scale from 0–10) they would be to recommend a hypothetical technology that could monitor back movements, provide feedback to improve posture, and decrease the likelihood of LBP. The answers are shown in Figure 4.

**Figure 5:**
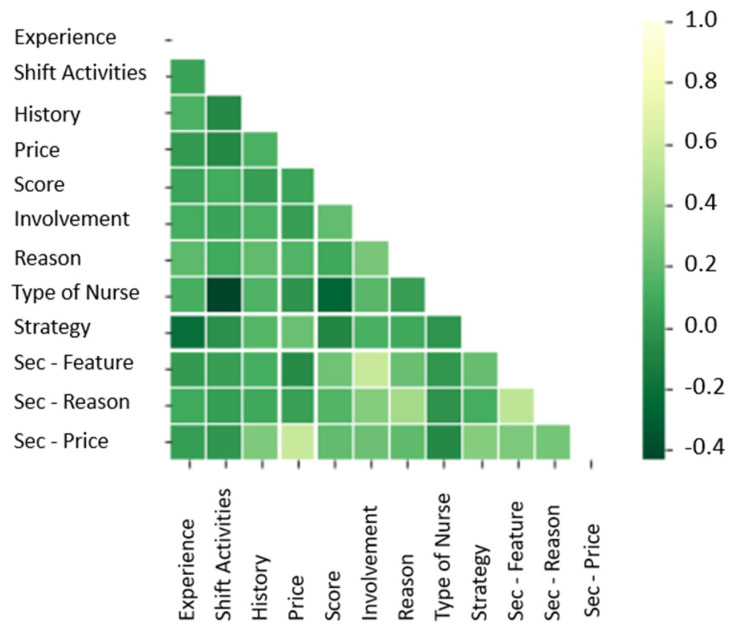
Correlation Matrix. The lighter a tile, the higher the correlation between the answers.

The data were organized to investigate correlations between some of the answers provided. Specifically, the correlations were computed between Experience (work experience), History (injury/pain history), shift activity, Price (price willing to pay), Score (how likely they would recommend the technology), Improvement (how likely they would like a technology to improve posture and reduce the likelihood of LBP), Reasons (reasons for the interest in the technology), Strategy (type of nurse, strategy/solution adopted to reduce LBP), Sec-Feature (interest in a second feature), Sec-Reason (the reason for the interest in a second feature), and Sec-Price (price willing to pay for a second feature). The heat map shown in Figure 5, with a gradient of colours from green to white, highlights the correlations between answers.

One of the goals was to know whether the population was interested in a potential technology for monitoring posture and movements and consequently discover if, with the selected questions, it was possible to highlight a pattern that led to such interest. From the heat map, it is possible to see that there is no strong correlation between the survey responses. The maximum correlation value reported between pairs of questions was 57%. The lack of linear correlation is insufficient to prove there is no pattern between answers. To further investigate and uncover the potential pattern, an RF method was adopted. Using RF and the method 80% and 20% train/test data, the first model was created with 32 nodes. The nodes selected were the minimum number of nodes required to create a high-performance model (e.g., over 80% accuracy).

The first model created was tested, and an accuracy of 91.66% was found using the RF model structure. In other words, with 11 given answers from a participant as input, the RF model predicted, with an accuracy of 91.66%, the price range that the participant was willing to pay. Furthermore, from the model, the weights of the answers were extrapolated. The weights represent the relevance of each question to predict the price. The feature relevance values are shown in Table 4.

For completeness, the RF model was built starting with randomly splitting the data using the CV with Stratified K-Fold [64]. This technique is broadly used, especially when the data set is considered small. CV with Stratified K-Fold gives a more robust model since it increases the information that the model acquires during training. In this scenario, the best number of nodes for the RF was nine, with a data structure randomly split in Train/Test of five Stratified K-Folds. The predictions from the model were compared with the survey test data, and for five-folds, the accuracy of the predictions was 81% ± 8%. All results from different sets of nodes (9, 32, and 100) are summarized in Table 5.

## 4. Discussion

The purpose of this study was to gain insights into nurses’ perceptions of LBP and whether they would consider the use of wearable monitoring technologies to reduce injury risk. Specifically, a survey to investigate if novel technologies could be introduced in the workplace was designed to determine the need for new technology among nurses and to understand which technology could be used based on how much workers are willing to pay for a solution.

The results confirmed that nurses perform high-risk tasks often during a shift. Furthermore, almost nine out of ten nurses surveyed were working in the presence of back pain or a diagnosed back condition. The most common strategy to reduce LBP risk was to adopt proper body mechanics during work, reported by more than one-third of respondents. This fits with the advice of occupational and public health strategies worldwide [65,66,67]. However, monitoring postures and movements while working is difficult. Throughout a shift, nurses may fatigue or be distracted by other more urgent tasks, placing them at risk of LBP through faulty movements or postures. Indeed, implementing lifting advice in the workplace has not led to a reduced occurrence of LBP [66].

Using wearable technology to monitor posture automatically and give feedback when workers assume high-risk movements could be helpful in preventing the occurrence of LBP. Providing feedback and promoting awareness of how workers move and the postures they use during daily activities can increase the effectiveness of guidelines put in place to prevent LBP. A recent pilot randomized controlled trial (N = 112) of individuals with chronic LBP showed that biofeedback from wireless movement sensors, combined with individualized rehabilitation, resulted in significant and sustained clinical improvements compared to standard treatment [68]. Another large-scale (N = 492) trial is underway to investigate the clinical effectiveness and economic efficiency of individualized cognitive functional therapy delivered with or without wearable biofeedback for patients with chronic LBP [69]. Similar gains could be made by implementing such a technology as a preventative measure.

Wearable technology has been shown to be useful in other conditions to drive behaviour change via biofeedback [70,71,72]. Biofeedback can help workers better understand their movements and postures and improve their voluntary control over automatic or involuntary processes [73]. Almost two-thirds of respondents indicated that they would be interested in a device that provided biofeedback about body alignment, bad postures, or strain risks. The use of a wearable device that can provide biofeedback regarding posture or lifting technique has the potential to reduce the risk of injury in this high-risk population. As a first step, using these technologies to study the links between posture or lifting technique and the risk of developing low back pain is also an intriguing opportunity. Until now, it was unknown whether a device like this would be of interest to this population. The results demonstrate that nurses are open to this type of technology, with half of those interested willing to pay less than CAD 50 and one-third willing to pay CAD 50–99 for the device. Given the cost of some wearable devices on the market today (such as IMUs), it is realistic that a device that could monitor basic postures and movements specific to nursing could be made within this price range [40]. Respondents were interested in a number of other features that could be added to such a device, including standing time, time spent in the same posture, posture while lifting, advice on correct postures, and recording of incorrect postures. However, adding more features did not significantly change the price nurses would be willing to pay for the device.

By adopting a machine learning technique, a pattern emerged that associates the price with the other answers. Forecasting human behaviour is highly unpredictable. An accuracy of 50% is often considered a good prediction [58,74]. The highest accuracy of 91.66% is inflated by Sec–Price inside the computation. Removing Sec–Price from the analysis, it is still interesting to see that features as the History of LBP and Score have a significant impact on the price (see Table 4). Furthermore, contrary to our assumptions, we observed that Experience, shift activities, and Type of nurse have a low impact on the price.

Most respondents were interested in a device that could document a back injury as work-related to assist with insurance claims and help better understand the cause of the injury. This has important implications both for insurance providers and employers and treatment interventions. Understanding the mechanism of injury may assist clinicians and patients in resolving the condition and avoiding future episodes.

### Limitations

This study is not without limitations. First, the recruitment was conducted via a purposive, nonprobabilistic sampling method. This may have led to bias in the sample selected to complete the survey. Therefore, it is not possible to assume that the findings are fully representative of the entire nursing population. Forty-four percent of the population surveyed had more than 10 years of work experience. This distribution of experience could also be a factor that impacted the acceptance of new wearable technology. The data collected in this study are descriptive in nature, and using an online design may be subject to response bias. Furthermore, the sample size was small (n = 64), which gave a confidence level of 90% with a margin of error of approximately 10%. Given that the proposed device was hypothetical in nature, the responses given regarding this device may not apply to the end-user technology developed for this population.

## 5. Conclusions

Nurses are a high-risk group for developing work-related LBP, as demonstrated by the high incidence of LBP and low back conditions in this sample. There are several options for wearable technology to assist in preventing LBP via monitoring and biofeedback. Findings from this study indicate that nurses are interested in these new approaches to monitoring movement and posture in the workplace to increase their awareness and prevent LBP by applying guidelines and procedures suggested from ergonomic studies. There is a potential for wearable technologies to prevent low back pain through timely and tailored feedback of movements and postures. Future studies should investigate the application of wearable technologies in the monitoring and prevention of low back pain in high-risk workplace settings.

## Figures and Tables

**Table 1:** Survey topics.

**Topic 1—Nurse demographics**	Gender; Type or regulated nurse; how long they have been a nurse and where; how often do they move patients.
**Topic 2—LBP & Back Issues-history**	If they have experienced LBP or other back issues.
**Topic 3—Strategies and solutions**	If they need a hypothetical new technology to prevent LBP and if they use a strategy or solution to mitigate or prevent LBP and, the reason for their choice
**Topic 4—Technology and solutions opinions**	A series of questions of which features they would need or dislike, how much would they pay for the technology described by the features selected in the previous questions and, how likely they will recommend the technology to a colleague. Short open questions with general recommendations about the topic.

**Table 2:** Demographics of survey participants.

	Description	Percentage
Gender	Female	78
	Male	19
	Not specified	3
Experience	More than 10 years	44
	3 to 10 years	25
	one to three years	11
	Less than one	20

**Table 3:** Suggestions by respondents of type of feedback.

Type of Feedback	List of Suggestions
Haptic-feedback based	Standing time, time spent in the same posture, body symmetry stair count, heart rate monitoring, mobility, calories burned, points for good postures, proper body mechanics, upper body twisting.
Visual and text-based	Lift techniques, posture while lifting, correct posture advice, video suggestions & information, incorrect postures recorded, the muscle used, statistics that can be reviewed with a physiotherapist, safety advice.

**Table 4:** Feature relevance values.

Features	Weight
Sec–Price	0.369618
Score	0.127149
History	0.115951
Reason	0.067282
Sec-reason	0.051463
Experience	0.048073
Strategy	0.047945
Involvement	0.045196
Shift activities	0.045089
Type of nurse	0.041400
Sec-Feature	0.040833

**Table 5:** Results from the RF model changing the numbers of nodes and data structures.

Model	Data Structures	Accuracy
RF-9 Nodes	80% Training-20% Testing	66.66%
RF-32 Nodes	80% Training-20% Testing	91.66%
RF-100 Nodes	80% Training-20% Testing	83.33%
RF-9 Nodes	CV & 5 Stratified K-Folds	81% ± 8%
RF-32 Nodes	CV & 5 Stratified K-Folds	79% ± 10%
RF-100 Nodes	CV & 5 Stratified K-Folds	79% ± 10%

## Data Availability

Data may be made available upon request.

## References

[1] Musculoskeletal Conditions. https://www.who.int/news-room/fact-sheets/detail/musculoskeletal-conditions.

[2] Hoy D., March L., Brooks P., Blyth F., Woolf A., Bain C., Williams G., Smith E., Vos T., Barendregt J. (2014). The Global Burden of Low Back Pain: Estimates from the Global Burden of Disease 2010 Study. Ann. Rheum. Dis..

[3] Bunzli S., Gillham D., Esterman A. (2011). Physiotherapy-Provided Operant Conditioning in the Management of Low Back Pain Disability: A Systematic Review. Physiother. Res. Int..

[4] Murphy P.L., Courtney T.K. (2000). Low Back Pain Disability: Relative Costs by Antecedent and Industry Group. Am. J. Ind. Med..

[5] Costs and Outcomes of Chiropractic Treatment for Low Back Pain. https://www.cadth.ca/costs-and-outcomes-chiropractic-treatment-low-back-pain-0.

[6] Crow W.T., Willis D.R. (2009). Estimating Cost of Care for Patients With Acute Low Back Pain: A Retrospective Review of Patient Records. J. Osteopath. Med..

[7] AWCBC ACATC: Statistics. http://awcbc.org/?page_id=14.

[8] Ehrlich G.E. (2003). Osteoarthritis Beginning with inflammation: Definitions and correlations. Bull. World Health Organ..

[9] Saraceni N., Kent P., Ng L., Campbell A., Straker L., O’Sullivan P. (2019). To Flex or Not to Flex? Is There a Relationship Between Lumbar Spine Flexion During Lifting and Low Back Pain? A Systematic Review With Meta-Analysis. J. Orthop. Sports Phys. Ther..

[10] Gallagher S., Marras W.S. (2012). Tolerance of the Lumbar Spine to Shear: A Review and Recommended Exposure Limits. Clin. Biomech..

[11] Nachemson A. (1963). The Influence of Spinal Movements on the Lumbar Intradiscal Pressure and on the Tensile Stresses in the Annulus Fibrosus. Acta Orthop. Scand..

[12] Adams M.A., Burton K., Bogduk N. (2006). The Biomechanics of Back Pain.

[13] Herman I.P. (2016). Physics of the Human Body; Biological and Medical Physics, Biomedical Engineering.

[14] Hamill J. (2006). Biomechanical Basis of Human Movement.

[15] Adams M.A. (2004). Biomechanics of Back Pain. Acupunct. Med..

[16] Gallagher S., Marras W.S., Litsky A.S., Burr D. (2005). Torso Flexion Loads and the Fatigue Failure of Human Lumbosacral Motion Segments. Spine.

[17] Knapik G.G., Marras W.S. (2009). Spine Loading at Different Lumbar Levels during Pushing and Pulling. Ergonomics.

[18] Jull G.A., Richardson C.A. (2000). Motor Control Problems in Patients with Spinal Pain: A New Direction for Therapeutic Exercise. J. Manip. Physiol. Ther..

[19] Hodges P.W., Richardson C.A. (1996). Inefficient Muscular Stabilization of the Lumbar Spine Associated With Low Back Pain: A Motor Control Evaluation of Transversus Abdominis. Spine.

[20] Bryan M., Hawson S. (2003). The Benefits of Pilates Exercise in Orthopaedic Rehabilitation. Tech. Orthop..

[21] Dillen L.R., Sahrmann S.A., Norton B.J., Caldwell C.A., McDonnell M.K., Bloom N. (2003). The Effect of Modifying Patient-Preferred Spinal Movement and Alignment during Symptom Testing in Patients with Low Back Pain: A Preliminary Report. Arch. Phys. Med. Rehabil..

[22] Hodder J.N., Holmes M.W.R., Keir P.J. (2010). Continuous Assessment of Work Activities and Posture in Long-Term Care Nurses. Ergonomics.

[23] Mital A. (1997). Recognizing Musculoskeletal Injury Hazards in the Upper Extremities and Lower Back. Occup. Health Saf..

[24] Herberts P., Kadefors R., Högfors C., Sigholm G. (1984). Shoulder Pain and Heavy Manual Labor. Clin. Orthop. Relat. Res..

[25] Hagberg M. (1984). Occupational Musculoskeletal Stress and Disorders of the Neck and Shoulder: A Review of Possible Pathophysiology. Int. Arch. Occup. Environ. Health.

[26] Westgaard R.H., Waersted M., Jansen T., Aaras A. (1986). Muscle Load and Illness Associated with Constrained Body Postures. Ergon. Work. Postures.

[27] Heliövaara M., Knekt P., Aromaa A. (1987). Incidence and Risk Factors of Herniated Lumbar Intervertebral Disc or Sciatica Leading to Hospitalization. J. Chronic Dis..

[28] Chaffin D.B. (1974). Human Strength Capability and Low Back. J. Occup. Med..

[29] Frymoyer J.W., Pope M.H., Costanza M.C., Rosen J.C., Goggin J.E., Wilder D.G. (1980). Epidemiologic Studies of Low-Back Pain. Spine.

[30] Andersson G.B.J. (1981). Epidemiologic Aspects on Low-Back Pain in Industry. Spine.

[31] Kumar S. (1990). Kumar Cumulative Load as a Risk Factor for Back Pain. Spine.

[32] Jones T., Kumar S. (2001). Physical Ergonomics in Low-Back Pain Prevention. J. Occup. Rehabil..

[33] Kumar S. (1999). Biomechanics in Ergonomics.

[34] WorkSafeBC. https://www.worksafebc.com/en/resources/health-safety/books-guides/does-your-back-hurt-a-guide-to-preventing-low-back-pain?lang=en&origin=s&returnurl=https%3A%2F%2Fwww.worksafebc.com%2Fen%2Fsearch%23q%3Ddoes%2520your%2520back%2520hurt%26sort%3Drelevancy%26f%3Alanguage-facet%3D%5BEnglish%5D.

[35] Delleman N.J., Dul J. (2007). International Standards on Working Postures and Movements ISO 11226 and EN 1005-4. Ergonomics.

[36] 14:00–17:00 ISO 11226:2000. https://www.iso.org/cms/render/live/en/sites/isoorg/contents/data/standard/02/55/25573.html.

[37] McAtamney L., Nigel Corlett E. (1993). RULA: A Survey Method for the Investigation of Work-Related Upper Limb Disorders. Appl. Ergon..

[38] Barim M.S., Sesek R.F., Capanoglu M.F., Drinkaus P., Schall M.C., Gallagher S., Davis G.A. (2019). Improving the Risk Assessment Capability of the Revised NIOSH Lifting Equation by Incorporating Personal Characteristics. Appl. Ergon..

[39] Ejupi A., Ferrone A., Menon C. Quantification of Textile-Based Stretch Sensors Using Machine Learning: An Exploratory Study. Proceedings of the 2018 7th IEEE International Conference on Biomedical Robotics and Biomechatronics (Biorob).

[40] Charry E., Umer M., Taylor S. (2011). Design and Validation of an Ambulatory Inertial System for 3-D Measurements of Low Back Movements. 2011 Seventh International Conference on Intelligent Sensors, Sensor Networks and Information Processing.

[41] BackTone Posture. Trainer. https://www.backtone.com.

[42] Introducing the Kinetic Reflex. https://www.wearkinetic.com/reflex-2/.

[43] Prana Science. http://prana.co/science/.

[44] Alex-Wearable Posture Tracker & Coach. https://www.avivahealth.com/products/alex.

[45] LifeBooster. https://lifebooster.ca/solutions/.

[46] Doss R., Robathan J., Abdel-Malek D., Holmes M.W.R. (2018). Posture Coaching and Feedback during Patient Handling in a Student Nurse Population. IISE Trans. Occup. Ergon. Hum. Factors.

[47] Bootsman R., Markopoulos P., Qi Q., Wang Q., Timmermans A.A. (2019). Wearable Technology for Posture Monitoring at the Workplace. Int. J. Hum. Comput. Stud..

[48] Papi E., Koh W.S., McGregor A.H. (2017). Wearable Technology for Spine Movement Assessment: A Systematic Review. J. Biomech..

[49] O’Sullivan K., O’Sullivan L., O’Sullivan P., Dankaerts W. (2013). Investigating the Effect of Real-Time Spinal Postural Biofeedback on Seated Discomfort in People with Non-Specific Chronic Low Back Pain. Ergonomics.

[50] Gao Y., Li H., Luo Y. (2015). An Empirical Study of Wearable Technology Acceptance in Healthcare. Ind. Manag. Data Syst..

[51] Kelley K. (2003). Good Practice in the Conduct and Reporting of Survey Research. Int. J. Qual. Health Care.

[52] Choi B.C.K., Pak A.W.P. (2004). A Catalog of Biases in Questionnaires. Prev. Chronic. Dis..

[53] Deming W.E. (1944). On Errors in Surveys. Am. Sociol. Rev..

[54] Seaborn: Statistical Data Visualization—Seaborn 0.10.0 Documentation. https://seaborn.pydata.org/.

[55] Pedregosa F., Varoquaux G., Gramfort A., Michel V., Thirion B., Grisel O., Blondel M., Prettenhofer P., Weiss R., Dubourg V. (2011). Scikit-Learn: Machine Learning in Python. J. Mach. Learn. Res..

[56] McKinney W. (2011). Pandas: A Foundational Python Library for Data Analysis and Statistics. Python High Perform. Sci. Comput..

[57] Stone M. (1974). Cross-Validatory Choice and Assessment of Statistical Predictions. J. R. Stat. Soc. Ser. B Methodol..

[58] Buskirk T.D. (2018). Surveying the Forests and Sampling the Trees: An Overview of Classification and Regression Trees and Random Forests with Applications in Survey Research. Surv. Pract..

[59] 12 York Street, 2nd Floor; Ottawa, O.K. 5S6; Canada Survey Sample Size Calculator. http://fluidsurveys.com/university/survey-sample-size-calculator/.

[60] Nursing Statistics. https://www.cna-aiic.ca/en/nursing-practice/the-practice-of-nursing/health-human-resources/nursing-statistics.

[61] Jang R., Karwowski W., Quesada P.M., Rodrick D., Sherehiy B., Cronin S.N., Layer J.K. (2007). Biomechanical Evaluation of Nursing Tasks in a Hospital Setting. Ergonomics.

[62] Yip Y. (2001). A Study of Work Stress, Patient Handling Activities and the Risk of Low Back Pain among Nurses in Hong Kong. J. Adv. Nurs..

[63] Weiner C., Alperovitch-Najenson D., Ribak J., Kalichman L. (2015). Prevention of Nurses’ Work-Related Musculoskeletal Disorders Resulting From Repositioning Patients in Bed: Comprehensive Narrative Review. Workplace Health Saf..

[64] Diamantidis N.A., Karlis D., Giakoumakis E.A. (2000). Unsupervised Stratification of Cross-Validation for Accuracy Estimation. Artif. Intell..

[65] Hogan D.A.M., Greiner B.A., O’Sullivan L. (2014). The Effect of Manual Handling Training on Achieving Training Transfer, Employee’s Behaviour Change and Subsequent Reduction of Work-Related Musculoskeletal Disorders: A Systematic Review. Ergonomics.

[66] Martimo K.-P., Verbeek J., Karppinen J., Furlan A.D., Takala E.-P., Kuijer P.P.F.M., Jauhiainen M., Viikari-Juntura E. (2008). Effect of Training and Lifting Equipment for Preventing Back Pain in Lifting and Handling: Systematic Review. BMJ.

[67] Verbeek J., Martimo K.P., Karppinen J., Kuijer P.P., Takala E.P., Viikari-Juntura E. (2012). Manual Material Handling Advice and Assistive Devices for Preventing and Treating Back Pain in Workers: A Cochrane Systematic Review. Occup. Environ. Med..

[68] Kent P., Laird R., Haines T. (2015). The Effect of Changing Movement and Posture Using Motion-Sensor Biofeedback, versus Guidelines-Based Care, on the Clinical Outcomes of People with Sub-Acute or Chronic Low Back Pain-a Multicentre, Cluster-Randomised, Placebo-Controlled, Pilot Trial. BMC Musculoskelet. Disord..

[69] Kent P., O’Sullivan P., Smith A., Haines T., Campbell A., McGregor A.H., Hartvigsen J., O’Sullivan K., Vickery A., Caneiro J. (2019). RESTORE—Cognitive Functional Therapy with or without Movement Sensor Biofeedback versus Usual Care for Chronic, Disabling Low Back Pain: Study Protocol for a Randomised Controlled Trial. BMJ Open.

[70] Pfeufer D., Gililland J., Böcker W., Kammerlander C., Anderson M., Krähenbühl N., Pelt C. (2019). Training with Biofeedback Devices Improves Clinical Outcome Compared to Usual Care in Patients with Unilateral TKA: A Systematic Review. Knee Surg. Sports Traumatol. Arthrosc..

[71] Kondo K., Noonan K.M., Freeman M., Ayers C., Morasco B.J., Kansagara D. (2019). Efficacy of Biofeedback for Medical Conditions: An Evidence Map. J. Gen. Intern. Med..

[72] Napier C., MacLean C.L., Maurer J., Taunton J.E., Hunt M.A. (2018). Real-Time Biofeedback of Performance to Reduce Braking Forces Associated With Running-Related Injury: An Exploratory Study. J. Orthop. Sports Phys. Ther..

[73] Dursun N., Dursun E., Kiliç Z. (2001). Electromyographic Biofeedback–Controlled Exercise versus Conservative Care for Patellofemoral Pain Syndrome. Arch. Phys. Med. Rehabil..

[74] Phan N., Dou D., Piniewski B., Kil D. (2016). A Deep Learning Approach for Human Behavior Prediction with Explanations in Health Social Networks: Social Restricted Boltzmann Machine (SRBM+). Soc. Netw. Anal. Min..

